# Recurrent Connections Might Be Important for Hierarchical Categorization

**DOI:** 10.3389/fnsys.2022.805990

**Published:** 2022-02-24

**Authors:** Narihisa Matsumoto, Yusuke Taguchi, Masaumi Shimizu, Shun Katakami, Masato Okada, Yasuko Sugase-Miyamoto

**Affiliations:** ^1^Human Informatics and Interaction Research Institute, National Institute of Advanced Industrial Science and Technology (AIST), Tsukuba, Japan; ^2^Graduate School of Science and Technology, University of Tsukuba, Tsukuba, Japan; ^3^Graduate School of Frontier Sciences, The University of Tokyo, Kashiwa, Japan

**Keywords:** visual category, visual cortex, short-term memory, deep learning, modeling

## Abstract

Visual short-term memory is an important ability of primates and is thought to be stored in area TE. We previously reported that the initial transient responses of neurons in area TE represented information about a global category of faces, e.g., monkey faces vs. human faces vs. simple shapes, and the latter part of the responses represented information about fine categories, e.g., facial expression. The neuronal mechanisms of hierarchical categorization in area TE remain unknown. For this study, we constructed a combined model that consisted of a deep neural network (DNN) and a recurrent neural network and investigated whether this model can replicate the time course of hierarchical categorization. The visual images were stored in the recurrent connections of the model. When the visual images with noise were input to the model, the model outputted the time course of the hierarchical categorization. This result indicates that recurrent connections in the model are important not only for visual short-term memory but for hierarchical categorization, suggesting that recurrent connections in area TE are important for hierarchical categorization.

## Introduction

Visual short-term memory is an important ability of primates. When primates see objects, the information about the objects is processed from the retina to the visual cortex in the brain. In the visual cortex, the object information is processed from V1 to area TE of the inferior temporal cortex (Mishkin et al., 1983). Visual short-term memory is thought to be stored in area TE (Sugase-Miyamoto et al., 2008) and the prefrontal cortex (Freedman et al., 2001). In area TE, some neurons respond to complex objects, faces, and so on and represent information about a global category, e.g., human vs. monkey vs. simple shapes, earlier than fine category information about faces, e.g., facial expression or identity (Sugase et al., 1999; Matsumoto et al., 2005a; Sugase-Miyamoto et al., 2014). In our previous study, we constructed a deep neural network (DNN) to compare information representation in each layer and information encoded by a neural population in area TE with a visual stimulus set that included human and monkey faces (Matsumoto et al., 2021). We found that the time course of hierarchical categorization could not be replicated with the DNN. Furthermore, global categorization occurred in the lower layers of the DNN. In this study, we hypothesize that visual short-term memory is retrieved from global to fine information of images *via* recurrent connections in area TE. To test this hypothesis, we constructed a combined model of a DNN, i.e., Xception net (Chollet, 2017), and a recurrent neural network, i.e., Hopfield model (Hopfield, 1982). The Hopfield model is known as an associative memory model (Anderson, 1972; Kohonen, 1972; Nakano, 1972). An associative memory model is considered a short-term memory model because it can store and retrieve original images from noise-degraded images. The combined model performed better for adversarial examples than using only the Xception net. The combined model also outputs the time course of hierarchical categorization. This indicates that recurrent connections in the Hopfield model are important for hierarchical categorization, suggesting that recurrent connections in area TE are important for such categorization.

## Materials and Methods

### Model

**FIGURE 1:**
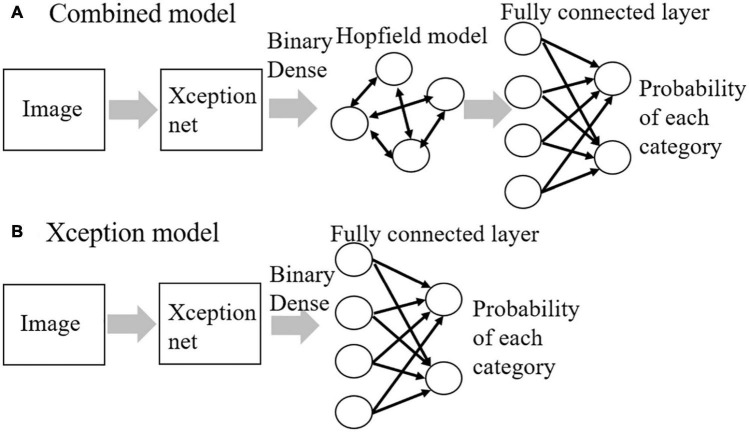
The model structures for **(A)** combined model and **(B)** Xception model.

We constructed our combined model consisting of an Xception net and a Hopfield model to investigate whether it can replicate the time course of hierarchical categorization (Figure 1A). Model parameters including weight values of the original Xception net were downloaded from https://github.com/keras-team/keras. The downloaded weight values were determined from images in the ImageNet database (Russakovsky et al., 2015). The weight values of the Xception net were fixed in this study. The top layer of the original Xception net is a fully connected layer that outputs the probability of each category. The fully connected layer was removed from the original Xception net, and the Hopfield model was inserted instead as a model of area TE. This was done because our previous studies showed that the information representation in fully connected layers of a DNN was similar to the representation in area TE (Matsumoto et al., 2021) and that an associative memory model was able to reproduce the neural activities of area TE (Matsumoto et al., 2005b). We compared the performance of the combined model with another model, i.e., the Xception model without the Hopfield model (Figure 1B). The inputs to the models were visual images (250 × 250 pixels, RGB color) and the outputs were the image category probabilities. In the learning phase, the weights of a binary dense layer (Hubara et al., 2016) and fully connected layers were learned using a backpropagation algorithm (Rumelhart et al., 1986) in both models, and weights of the Hopfield model were learned by the Storkey rule (Storkey, 1997) or the covariance rule for the combined model. In the test phase, adversarial examples generated from the learned images or learned images with Gaussian noise were given as input to the combined model. The code of the model was written using TensorFlow (Abadi et al., 2015) and Keras (Chollet, 2015).

**FIGURE 2:**
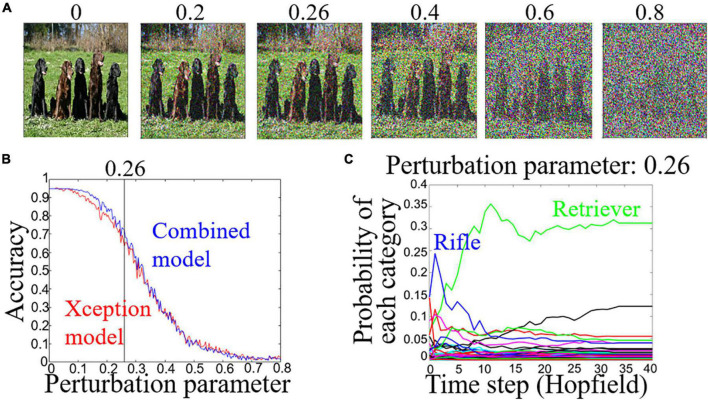
**(A)** Adversarial examples for perturbation parameter. The original image is taken from the ImageNet database. **(B)** Accuracy in estimating correct category for perturbation parameters of the Xception model (red line) and combined model (blue line). **(C)** Time course for the probability of each category for Retriever image **(A)** at perturbation parameter 0.26.

The Hopfield model consists of *N* neurons. The internal potential of neuron *i* at time *t* is denoted as *h*_*i*_(*t*) and updated as given by the following equation,


(1) $$h_{i}\,\left(t\right)=\sum_{j\neq i}^{N}J_{i\,j}\,s_{j}\,\left(t\right),$$

where *J*_*ij*_ denotes a synaptic weight of recurrent connection from neuron *j* to neuron *i*, and *s*_*j*_(*t*) denotes the state of neuron *j* at time *t* (*s*_*j*_(*t*) = {1, −1}):


(2) $$s_{j}\,\left(t+1\right)=\text{sign}\,\left(h_{j}\,\left(t\right)\right),$$

where sign[*h*_*j*_(*t*)] is a sign function: if *h*_*j*_(t) ≥ 0, sign[*h*_*j*_(*t*)] = 1: otherwise, sign[*h*_*j*_(*t*)] = −1. A feature vector of the binary dense layer was used as the memory pattern ξ^μ^ for each image and set as an initial state, *s*(0), of the Hopfield model. The weight was determined by the Storkey rule (results are shown in Figure 2),


(3) $$J_{i\,j}^{\nu }=J_{i\,j}^{\nu -1}+\frac{1}{N}\,\xi_{i}^{\nu }\,\xi_{j}^{\nu }-\frac{1}{N}\,\xi_{i}^{\nu }\,f_{j\,i}^{\nu }-\frac{1}{N}\,\xi_{j}^{\nu }\,f_{i\,j}^{\nu },$$

where ν={1,…,μ}, *J_*ij*_ = J_*ij*_^μ^*, and *f_*ij*_^ν^* obeys:


(4) $$f_{i\,j}^{\nu }=\sum_{k\neq i,j}^{N}J_{i\,k}^{\nu -1}\,\xi_{k}^{\nu }.$$

**FIGURE 3:**
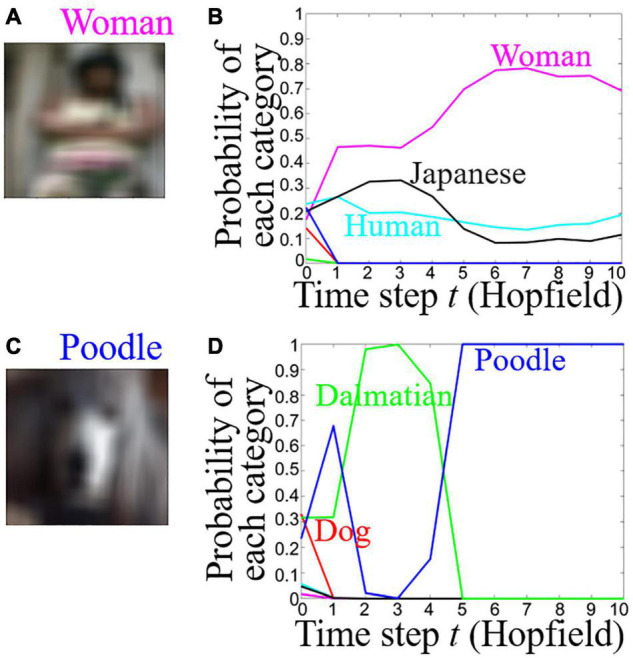
Time course of the probability of each category for Woman and Poodle images with Gaussian noise. **(A,C)** Woman and Poodle images with Gaussian noise. **(B,D)** Time course of the probability of each category for Woman and Poodle images. Cyan: Human, magenta: Woman, black: Japanese, red: Dog, green: Dalmatian, blue: Poodle.

The weight *J*_*ij*_ was also determined by the covariance rule (results are shown in Figure 3),


(5) $$J_{i\,j}=\frac{1}{N}\,\sum_{\mu }^{p}\left(\xi_{i}^{\mu }-m\right)\,\left(\xi_{j}^{\mu }-m\right),$$

where *m* is the average of *ξ_*i*_^μ^*.

## Results

### Adversarial Examples

We tested whether our combined model can retrieve the correct category of images from noise-degraded images, i.e., adversarial examples. Adversarial examples were generated using VGG16 (Simonyan and Zisserman, 2014) and the fast gradient sign method (FGSM) (Goodfellow et al., 2014). We changed a perturbation parameter to obtain different amounts of noise (Figure 2A). In the learning phase, the weights of the binary dense layers and fully connected layers were learned from the 250 images by using the backpropagation algorithm. The weights of the Hopfield model (*N* = 5,000) were learned using the Storkey rule (Storkey, 1997) with 250 original images of 50 categories (Supplementary Table 1) taken randomly from the ImageNet database. In the test phase, the largest difference between the accuracies of the estimating categories of adversarial examples for the combined model and the Xception model was 9.2%, i.e., the accuracies were 72.4% (combined model) and 63.2% (Xception model), at the perturbation parameter 0.26 (Figure 2B). At the perturbation parameter 0.26, the combined model outputted the Rifle category at *t* = 0 for the image in Figure 2A and then outputted the Retriever category (Figure 2C). In other words, the model has an error-correcting ability of an associative memory model. At the perturbation parameter 0.26, the number of adversarial examples for each model performance is shown in Table 1.

**TABLE 1:** Number of adversarial examples classified by performance for the Xception model and the combined model at the perturbation parameter 0.26.

	Xception: correct	Xception: incorrect
Combined: correct	148	34
Combined: incorrect	59	9

### Images With Gaussian Noise

To examine whether the hierarchical categorizations were observed in the combined model, the combined model was tested using images with Gaussian noise. In the learning phase, the weights of the binary dense layers and fully connected layers were learned from the 30 original images of six categories (Human, Woman, Japanese, Dogs, Dalmatian, and Poodle) by using the backpropagation algorithm. The weights of the Hopfield model (*N* = 2,048) were learned using the covariance rule with 20 original images of four categories (Woman, Japanese, Dalmatian, and Poodle). Images of super-categories, i.e., Human and Dog,

were not learned in the Hopfield model. In the test phase, the learned images with Gaussian noise (mean: 0, variance: 0.1, size: 15 × 15 pixels) (Figures 3A,C) were given as input to the combined model. The model outputted the probability of each category at each time step. When a Woman or Poodle image with Gaussian noise (Figures 3A,C) was presented to the combined model, the model initially responded with the Human or Dog category, then responded with the correct category, i.e., Woman or Poodle (Figures 3B,D). The Hopfield model did not process information at the initial time step, *t = 0*. Therefore, the combined model was the same as the Xception model at only *t = 0*. The sum of the probability of each category was 1. At the initial time step *t* = 0, multiple categories had small probabilities, so the difference between Dog and Dalmatian became small. At *t* = 10 only a few categories had values of probability, and therefore, the difference among the categories became large. In Figure 3B, the output was Human (super-category) at *t = 0*, followed by Woman (sub-category). In Figure 3D, the output was Dog (super-category) at *t = 0*, followed by Poodle (sub-category), then Dalmatian, and finally Poodle again. In other words, the combined model has an error-correcting ability of an associative memory model as shown in the previous paragraph. Two of the three images that were assigned the correct category had this trend of hierarchical categorizations.

**FIGURE 4:**
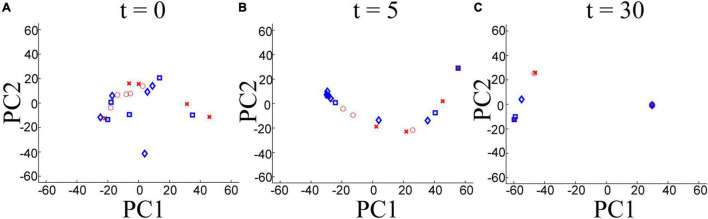
Two-dimensional space of state vectors of Hopfield model obtained by principal component analysis (PCA) at **(A)** *t* = 0, **(B)** *t* = 5, and **(C)** *t* = 30. Red circles: Woman, red crosses: Japanese, blue squares: Dalmatian, blue diamonds: Poodle.

To understand the temporal behavior of the Hopfield model, we projected the neuronal states into this model, i.e., 2,048-dimensional vectors, for 20 images into a two-dimensional space by principal component analysis (PCA) (Matsumoto et al., 2005a), as shown in Figure 4. The horizontal and vertical axes indicate the first and second principal components (PC1, PC2). The red points indicate Woman or Japanese. The blue points indicate Dalmatian or Poodle. At *t* = 0, the distributions for state vectors of Dalmatian and Poodle, and Woman and Japanese overlapped (Figure 4A). At *t* = 5, many state vectors for Dalmatian and Poodle were projected into the left side of Figure 4B, and most state vectors for Japanese were projected into the right side of Figure 4B. At *t* = 30, there were four clusters. A cluster contained the vectors of Woman and Japanese (Figure 4C). The others contained all four categories, i.e., Woman, Japanese, Dalmatian, and Poodle. Therefore, different categories were encoded in a different time course with the Hopfield model.

## Discussion

We constructed a model that combined an Xception net and a Hopfield model to investigate whether it can replicate the time course of a hierarchical categorization. The combined model for adversarial examples performed better than the Xception model. The combined model also outputted different categories during the time course. These results indicate that recurrent connections in the Hopfield model are important not only for short-term memory but also for hierarchical categorization, suggesting that recurrent connections in area TE are important for hierarchical categorization.

In our previous study, we showed that the behavior of an associative memory model was qualitatively similar to that of neurons in area TE (Matsumoto et al., 2005b). The model we constructed for that study used random bit patterns not visual images as input. In another study, we constructed a DNN, i.e., AlexNet (Krizhevsky et al., 2012), to compare the information represented in each layer and the information encoded by a neural population in area TE with a visual stimulus set that included human and monkey faces (Matsumoto et al., 2021). Thus, the representation in the fully connected layers of AlexNet most resembled the representation of TE neurons for human and monkey faces. Studies have suggested that recurrent processing is important for visual recognition (Spoerer et al., 2017; Kar et al., 2019). These models consist of recurrent connections in all layers, and each layer is not a Hopfield model. In a combined model which consisted of a DNN and a recurrent network, e.g., long short-term memory (LSTM) in Koo et al. (2019), to output hierarchical categories, a feature vector from top to bottom layer was given as input to LSTM at each time step. The feature vector in the top layer was inputted to LSTM at *t* = 0, the vector in the second-top layer was inputted to LSTM at *t* = 1. Therefore, the feature vectors in all layers should be stored in the memory. In our combined model a feature vector from a single layer of the Xception net was given as input to the Hopfield model at initial time step *t* = 0. The vector was updated by recurrent connections of the Hopfield model. Therefore, the structures of our combined model and the combined model of Koo et al. (2019) are different, and the structure of our model requires less memory consumption than that of the model of Koo et al. (2019). In our combined model, we added recurrent connections only to the Hopfield model layer to investigate whether recurrent processing in area TE is important for hierarchical categorization. We considered the Hopfield model as modeling for area TE in the higher visual cortex. The fully connected layers in our model were considered to be the prefrontal cortex or other higher brain areas that judge categories of visual images. Thus, our model can retrieve hierarchical categorical information from noise-degraded images and be considered as a model for short-term memory.

## Data Availability Statement

The raw data supporting the conclusions of this article will be made available by the corresponding author, NM (xmatumo@ni.aist.go.jp), upon reasonable request.

## Author Contributions

NM, MO, and YS-M designed the research and discussed the data. NM, YT, MS, and SK conducted the modeling. NM wrote the draft of the article. MO and YS-M revised the manuscript. All authors approved the final version of the manuscript.

## Conflict of Interest

YT is currently employed by the company IBM Japan. The remaining authors declare that the research was conducted in the absence of any commercial or financial relationships that could be construed as a potential conflict of interest.

## Publisher’s Note

All claims expressed in this article are solely those of the authors and do not necessarily represent those of their affiliated organizations, or those of the publisher, the editors and the reviewers. Any product that may be evaluated in this article, or claim that may be made by its manufacturer, is not guaranteed or endorsed by the publisher.
