# Supplementary material for: Recurrent Connections Might Be Important for Hierarchical Categorization
Source: Front Syst Neurosci. 2022 Feb 24;16:805990. doi: 10.3389/fnsys.2022.805990 (PMC8911877; doi:10.3389/fnsys.2022.805990)
Supplement: Supplementary file 1 [file Table_1.docx]

Supplementary Material

# Supplementary Table

| pencilsharpener- | cuirass- |
| --- | --- |
| carton- | stupa-tope- |
| mortaroard- | Newtoniantelescope-Newtonianreflector- |
| shaver-electricshaver-electricrazor- | dalmatian-coachdog-carriagedog- |
| flat-coatedretriever- | roin-Americanroin-Turdusmigratorius- |
| paddle-oatpaddle- | Komododragon-Komodolizard-dragonlizard-giantlizard-Varanuskomodoensis- |
| toiletseat- | snorkel- |
| cominationlock- | aseall- |
| drake- | chiffonier-commode- |
| inder-ring-inder- | rainarrel- |
| tale-tennistale-ping-pongtale-pingpongtale- | pirate-pirateship- |
| seasnake- | ison- |
| slothear-Melursusursinus-Ursusursinus- | powerdrill- |
| hermitcra- | greyfox-grayfox-Urocyoncinereoargenteus- |
| guillotine- | three-toedsloth-ai-Bradypustridactylus- |
| spidermonkey-Atelesgeoffroyi- | marima-xylophone- |
| sportscar-sportcar- | assaultrifle-assaultgun- |
| safetyrazor- | puffer-pufferfish-lowfish-gloefish- |
| fireell- | woodrait-cottontail-cottontailrait- |
| fryingpan-frypan-skillet- | hipflask-pocketflask- |
| milkcan- | limousine-limo- |
| cockroach-roach- | flatworm-platyhelminth- |
| drillingplatform-offshorerig- | jigsawpuzzle- |
| dowitcher- | treefrog-tree-frog- |
| ucket-pail- | ruffedgrouse-partridge-Bonasaumellus- |

**Supplementary Table 1.** The name of 50 categories.
